# Real-Time Human Pose Estimation and Gesture Recognition from Depth Images Using Superpixels and SVM Classifier

**DOI:** 10.3390/s150612410

**Published:** 2015-05-26

**Authors:** Hanguen Kim, Sangwon Lee, Dongsung Lee, Soonmin Choi, Jinsun Ju, Hyun Myung

**Affiliations:** 1 Urban Robotics Laboratory (URL), Dept. Civil and Environmental Engineering, Korea Advanced Institute of Science and Technology (KAIST), 291 Daehak-ro, Yuseong-gu, Daejeon 305-338, Korea; E-Mails: sskhk05@kaist.ac.kr (H.K.); lsw618@gmail.com (S.L.); 2 Image & Video Research Group, Samsung S1 Cooperation, 168 S1 Building, Soonhwa-dong, Joong-gu, Seoul 100-773, Korea; E-Mails: dslee.lee@samsung.com (D.L.); soonmin.choi@samsung.com (S.C.); jinsun.ju@samsung.com (J.J.)

**Keywords:** human pose estimation, gesture recognition, depth information, low-cost platform

## Abstract

In this paper, we present human pose estimation and gesture recognition algorithms that use only depth information. The proposed methods are designed to be operated with only a CPU (central processing unit), so that the algorithm can be operated on a low-cost platform, such as an embedded board. The human pose estimation method is based on an SVM (support vector machine) and superpixels without prior knowledge of a human body model. In the gesture recognition method, gestures are recognized from the pose information of a human body. To recognize gestures regardless of motion speed, the proposed method utilizes the keyframe extraction method. Gesture recognition is performed by comparing input keyframes with keyframes in registered gestures. The gesture yielding the smallest comparison error is chosen as a recognized gesture. To prevent recognition of gestures when a person performs a gesture that is not registered, we derive the maximum allowable comparison errors by comparing each registered gesture with the other gestures. We evaluated our method using a dataset that we generated. The experiment results show that our method performs fairly well and is applicable in real environments.

## 1. Introduction

Human pose estimation and gesture recognition are attractive research topics in computer vision and robotics owing to their many applications, including human computer interaction, game control and surveillance. The release of low-cost depth sensors, such as Microsoft Kinect for Xbox 360 and ASUS Xtion, has provided many important benefits to these research areas [1]. Kinect for Xbox 360 and Xtion are RGB-D (red, green, blue and depth) sensors that obtain depth information by structured light technology [2]. The structured light sensors infer the depth values by projecting an infrared light pattern onto a scene and analyzing the distortion of the projected light pattern. However, these sensors are limited to indoor use, and their low resolution and noisy depth information make it difficult to estimate human poses from depth images. Many human pose estimation methods use a GPU (graphic processing unit) to increase the frame rate and the performance [3–6]. These methods shows remarkable performance, but it is difficult to operate the algorithms on low-cost systems, such as embedded boards or mobile platforms. Other methods that do not use GPUs show low frame rates [7,8], and some cannot even run in real time [9]. Moreover, model-based approaches require model calibration before pose estimation [7,8].

Human pose estimation methods can be classified into two categories: model-based and learning-based approaches. In model-based approaches, prior knowledge of a human body model is required, and the human pose is estimated by inverting the kinematics or solving optimization problems. Grest *et al.* exploit the iterative closest point (ICP) approach with a body model to track a human pose initialized by a hashing method [8]. Siddiqui *et al.* use the Markov chain Monte Carlo (MCMC) framework with head, hand and forearm detectors to fit a body model [10]. Zhang *et al.* introduced a generative sampling algorithm with a refinement step of local optimization with a 3D body model for body pose tracking [3]. In learning-based approaches, however, a human body model is not considered, but human poses are directly estimated from input images with various machine learning algorithms. Shotton *et al.* present two different methods for human pose estimation [4]. The methods are based on a random forest trained on a large amount of synthetic human depth image data. One of the methods uses a per-pixel classification method, where each pixel on a human body is classified by the trained classification random forest. The other method predicts joint position by using a regression random forest. Each pixel on the human body directly votes on all of the joint positions. The classified pixels or the joint position votes are aggregated to estimate joint points by a mean shift. Hernández-Vela *et al.* extended the per-pixel classification method of Shotton *et al.* [4] using graph-cut optimization [5]. The graph-cut is an energy minimization framework, and it has been widely used in image segmentations.

Various methods are currently used for gesture recognition, including RGB-D sensor-based methods and other sensor-based (e.g., inertial measurement unit, electromyography, virtual reality gloves, *etc.*) methods [11]. Since the launch of Kinect for Xbox 360, many studies on gesture recognition use the skeleton information provided by Kinect for Xbox 360 [12,13] or directly use depth information [14,15]. However, with most of the algorithms, registration of arbitrary gestures that users may perform is not easy, because most of the algorithm use machine learning-based approaches that require a training process [13,16]. Furthermore, the recognition rate is easily affected by environmental changes [17].

Gesture recognition methods can be divided into two categories: matching-based and machine learning-based approaches. Wu *et al.* proposed a matching-based method that uses dynamic time-warping to identify users and recognize gestures with joint data from Kinect for Xbox 360 [12]. Megavannan *et al.* also proposed a matching-based algorithm that uses the motion dynamics of an object from the depth difference and average depth information [14]. The performance of matching-based approaches is easily affected by external noise or environmental changes, but training data and training phase are not required. The machine learning-based methods require training data and a training phase to generate classifiers, but they are more robust to noise and environmental changes than matching-based methods. Biswas *et al.* presented a method wherein SVMs are trained to classify gestures with depth difference information [13]. Sigalas *et al.* proposed an upper-body part tracking method and a gesture recognition method that combines a multi-layer perceptron and radial basis function neural networks [18].

In this paper, we propose human pose estimation and gesture recognition algorithms that use only depth information for robustness to environmental and lighting changes. The proposed algorithms are designed to be operated on low-cost systems, such as embedded boards and mobile platforms, without exploiting GPUs. Our pose estimation method is based on a per-pixel classification method where each pixel on the human body is classified into a body part. We reduce the computation time of body part classification by using superpixels. The proposed human pose estimation method can estimate human poses instantly without a calibration process, allowing the system to be used with any subject immediately. In the proposed gesture recognition method, the gesture registration process is simple, and gestures can be recognized regardless of motion speed by using key frame extraction. The proposed gesture recognition method is robust to environmental or lighting changes, as it uses only pose information, and our method can cover various motions for a single gesture by adapting the Mahalanobis distance [19] in comparing input motions with the registered gesture.

The remainder of this paper is organized as follows: Section 2 explains our human pose estimation method. Section 3 describes the proposed gesture recognition method. The proposed algorithms are evaluated through experiments in Section 4. Finally, a conclusion and directions for future work are provided in Section 5.

## 2. Human Pose Estimation

**Figure 1:**
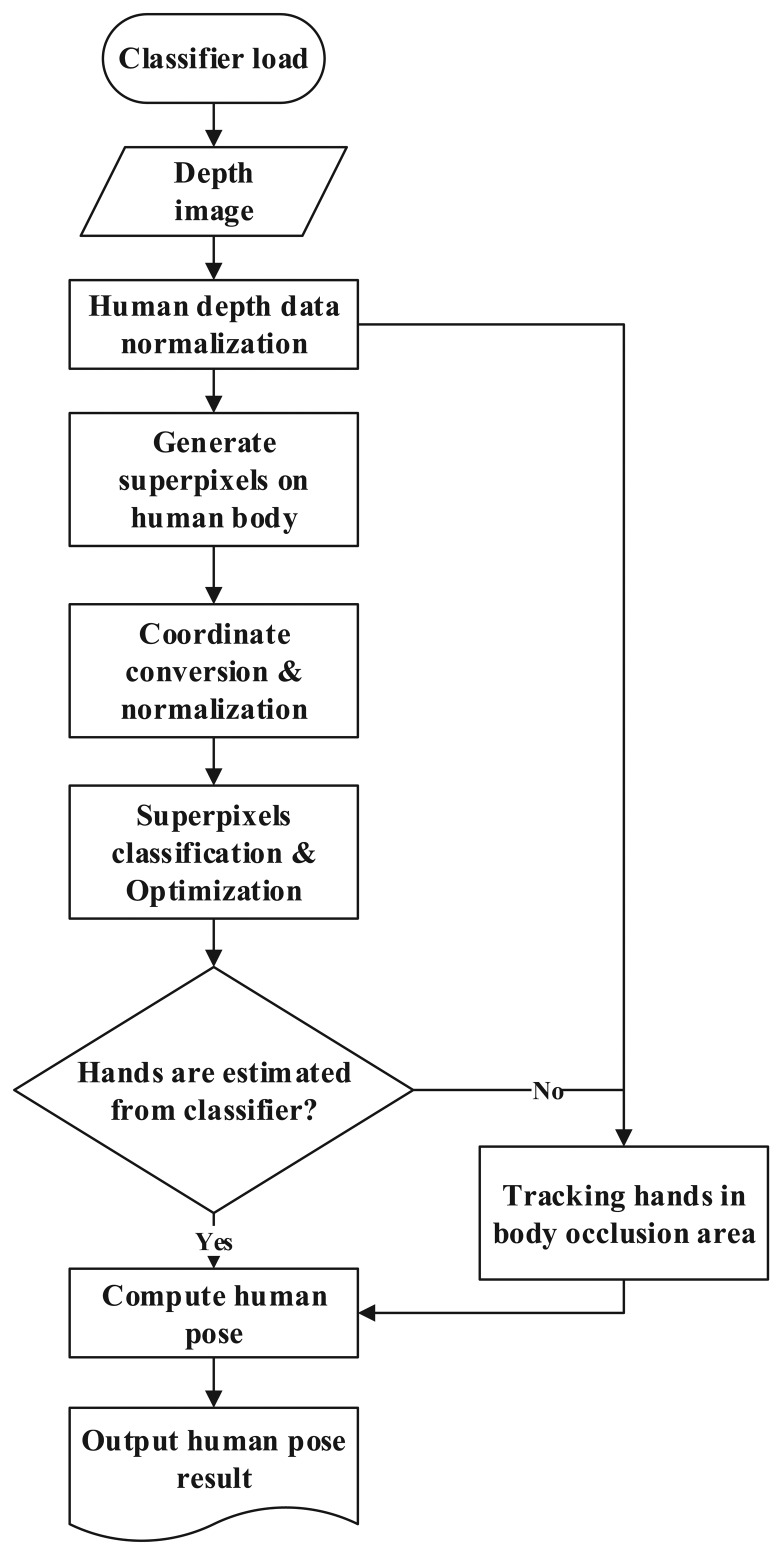
Flow diagram of the proposed human pose estimation method.

The proposed human pose estimation method is based on an SVM (support vector machine) and superpixels. Our pose estimation method predicts 15 joint positions of the human body: head, neck, torso, L/R (left/right) shoulders, L/R elbows, L/R hands, L/R hips, L/R knees and L/R feet. A flow diagram of the proposed pose estimation algorithm is presented in Figure 1. The proposed system extracts background-subtracted human depth ROIs (regions of interest), ensuring that each extracted ROI contains a human body without occlusion. Once a human body ROI is extracted from a depth image, the depth values of the pixels on the human body are normalized. The normalization process starts from finding the minimum and maximum depth values in the extracted ROI image. Then, the depth values are linearly mapped into 16-bit scale by using the minimum and maximum depth values. By normalization of depth values in the extracted ROI image, the ROI has the same depth value distribution regardless of the distance from the depth sensor. Furthermore, the normalization process stretches the range of the depth values in the ROI, so that small pixel differences in depth images become more distinguishable. After normalization, superpixels are generated on the human body using SLIC (simple linear iterative clustering) [20] for speeding up computation time. After superpixels are generated, the origin of *x*, *y* coordinates of superpixels to the central moment of the human body is to keep the coordinate points consistent regardless of the distance between the depth sensor and the human body, which is beneficial to the performance of SVM. The converted superpixels are then scaled to a predefined body size depending on the depth values of the human body. The processed superpixels are classified by a trained SVM classifier into one of the body parts, and the falsely-classified superpixels are removed in the optimization process. From the classified superpixels, the positions of the 15 joints are estimated. When hands are situated on or over the torso, they are not estimated from the classifier. In this case, the hands are tracked in the torso area using a Kalman filter. The overall procedures are presented in Figure 1.

### 2.1. Superpixel Feature Generation

**Figure 2:**
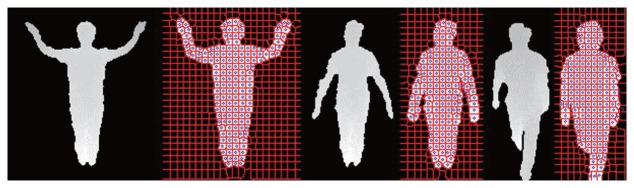
Results of superpixel generation on human bodies. The blue points on the human bodies are the generated superpixels.

Our pose estimation method uses per-pixel classification without prior human model information. However, a great deal of time is required to classify all of the pixels in a human ROI. To address this problem, instead of classifying all of the pixels in the human ROI, superpixels are generated on the human body, and then, the generated superpixels are classified into one of the body parts. This process tremendously reduced the amount of time required for body part classification. For the generation of superpixels, the SLIC (simple linear iterative clustering) superpixels method [20] is exploited. According to [20], the superpixels algorithm groups pixels into perceptually-meaningful atomic regions. The SLIC superpixels method adopts k-mean clustering to generate superpixels. The original superpixel algorithm iteratively clusters the pixels with similar color intensity within the user-defined search space. The proposed algorithm, however, generates a constant number of superpixels by using the depth values, which is to obtain the pose estimation within constant computation time regardless of the distance between the depth sensor and the human body. In this paper, the number of superpixels was empirically adjusted. The proposed method helps to keep the computation time for the human pose estimation almost constant. The exemplary results of superpixel generation on the human body are shown in Figure 2.

As the origin of the *x*, *y* coordinates in the human body ROI is the upper left vertex of the ROI and the width and the height of the ROI change with respect to the human pose, the *x*, *y* coordinates of the same body part vary with the human pose. To reduce these variations of the *x*, *y* coordinates, the origin of the *x*, *y* coordinates of superpixels is translated to the central moment of the human body. In addition to the coordinate conversion, the variation of the *x*, *y* coordinates of the superpixels caused by the distance change between the human body and the depth sensor should be considered. The variation of the *x*, *y* coordinates from the distance change is mitigated by multiplying the *x*, *y* coordinates by the scale factor *S* computed from the following equation:

(1) $$S=\frac{D_{\text{body}}}{D_{\text{ref}}}$$

where *D_body_* and *D_ref_* denote the average depth of the human body and the reference depth value, respectively. Equation (1) is derived on the basis of the relationship between the distance and the height of objects in an image being inverse-linear. Equation (1) means that the human body is placed at the reference distance *D_ref_*. Based on the specification of Kinect for Xbox 360 or ASUS Xtion Pro [21], the *D_ref_* can be set between 2000 and 4000 mm, where depth sensors give less noisy depth values and human body size is appropriate in the images.

#### 2.1.1. Pose Estimation

**Figure 3:**
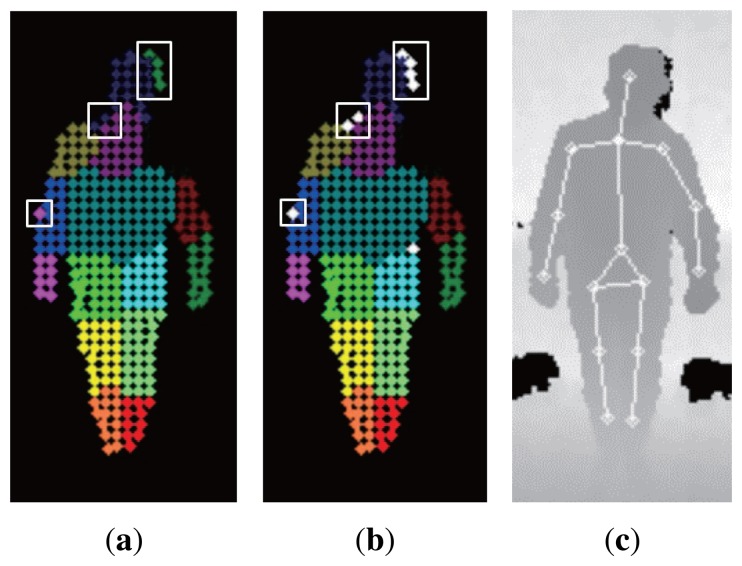
An example of: (**a**) superpixel classification; (**b**) optimization; (**c**) pose estimation. Misclassified superpixels removed in the optimization process are indicated by white rectangles.

The joint positions are estimated from the classified superpixels. Our human pose estimation method uses the SVM for classification of superpixel features. The SVM classifier learns from the training data that have been created by a motion capture system, and the performance of the classifier is verified by the ground truth data that have been also produced by the motion capture system. The motion capture system used in this paper will be explained in the Experiments Section. If there are some misclassified superpixels, the estimated joint positions may result in wrong positions. To prevent this, a misclassified superpixel is removed in the optimization process. When every superpixel is correctly classified, the superpixels that belong to the same body part are clustered on the corresponding body part. From this, we can assume that the misclassified superpixels are located far from the cluster of the corresponding body part. Therefore, by measuring the distance between the same body part superpixels, the misclassified superpixels can be identified and removed. We define *B* = {*B*_1_, *B*_2_,…, *B_i_*,…, *B_N_*}, a vector for body part labels whose components *B_i_* indicate a certain body part label, *P* = {*P*_1_, *P*_2_,…, *P_j_*, …, *P_M_*}, a set of the generated superpixels, and *b* = {*b*_1_, *b*_2_, …, *b_j_*, …, *b_M_*}, a vector for labels of the superpixels whose components *b_j_* specify the body part label assigned to a superpixel *P_j_*. Algorithm 1 shows the detailed procedure for removing the mislabeled superpixels. For a superpixel *P_j_* that belongs to a body part *B_i_*, *meanDist_B_i__*_,_*_P_j__*, the mean distance to the other superpixels, classified as *B_i_*, is computed. For the body part *B_i_*, *meanDist_B_i__*, the mean of *meanDist_B_i_,P_j__*, is computed. After computing the *meanDist_B_i_,P_j__* and *meanDist_B_i__*, every *meanDist_B_i__*_,_*_P_j__* is compared with *meanDist_B_i__*. If *meanDist_B_i_,P_j__* is bigger than *meanDist_B_i__*, the superpixel *P_j_* will be removed, because the superpixel *P_j_* is situated away from the cluster of superpixels classified as *B_i_*. The overall procedures can be found in Algorithm 1. Figure 3a,b present examples of misclassified superpixels and the optimized label results, respectively.



**Algorithm 1** Removal of mislabeled superpixels
1:**for** each body part label *B_i_* ∈ *B*
**do**2: **for** each superpixel *P_j_*(*b_j_* = *B_i_*) **do**3:  *meanDist_B_i_,P_j__* ← compute the mean distance to *P_k_*((*b_k_* = *B_i_*)&(*j* ≠ *k*))4: **end for**5: *meanDist_B_i__* ← compute the mean of *meanDist_B_i_,P_*6:**end for**7:**for** each body part label *B_i_* ∈ *B*
**do**8: **for** each superpixel *P_j_*(*b_j_* = *B_i_*) **do**9:  **if**
*meanDist_B_i_,P_j__* > *meanDist_B_i__*
**then**10:   *b_j_* ← (none)11:  **end if**12: **end for**13:**end for**


**Figure 4:**
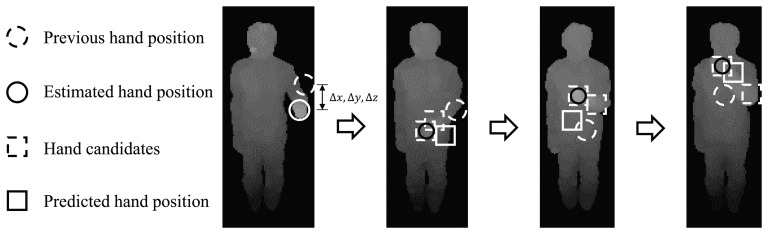
Example of the measurement update step when the hand occludes the torso. The hand tracker extracts the depth measurements for hand candidates. The final hand position is estimated by the depth measurement with the smallest Mahalanobis distance from the previous hand position.

After removing the misclassified superpixels, each joint position of *B_i_* is estimated as the central moment of the superpixels labeled as the corresponding body part *B_i_*. An example of a pose estimation result is shown in Figure 3c. However, when the hands occlude the torso, none of the superpixels are classified as hands. To solve this problem, a hand tracker is designed to estimate the hand position, even when the hand information is not provided by the classifier. The hand tracker is designed based on the Kalman filter. The Kalman filter usually consists of two steps. One is the state prediction step, and the other is the measurement update step, which are calculated at every frame in the background process. In the state prediction step, the state is estimated based on the previous hand position and the hand position difference, i.e., Δ*x*, Δ*y* and Δ*z*. In the measurement update step, the hand tracker extracts the depth measurements for hand candidates within the ROI (region of interest), which is calculated from the previous hand position and Δ*x*, Δ*y* and Δ*z*. The hand position is updated by the depth measurement with the smallest Mahalanobis distance from the previous hand position. If the hand position can be acquired from the classification results, the hand position is corrected by the classification results. Otherwise, the result from the Kalman filter is finally used as the hand position. The exemplary measurement update procedure is shown in Figure 4 in case the hand occludes the torso. The rationale for applying the linear Kalman filter as a hand tracker is as follows. The first reason is that the hand movements are continuous. Therefore, the hand position can be predicted by using the previous hand position and its position difference. The second reason is that the hand movement being faster than the operation speed of the overall algorithm (in our experimental setting, the overall algorithm runs at 15 frames per second) was not considered. This means that the hand tracker can track the general hand movements continuously within the operation speed.

## 3. Gesture Recognition

**Figure 5:**
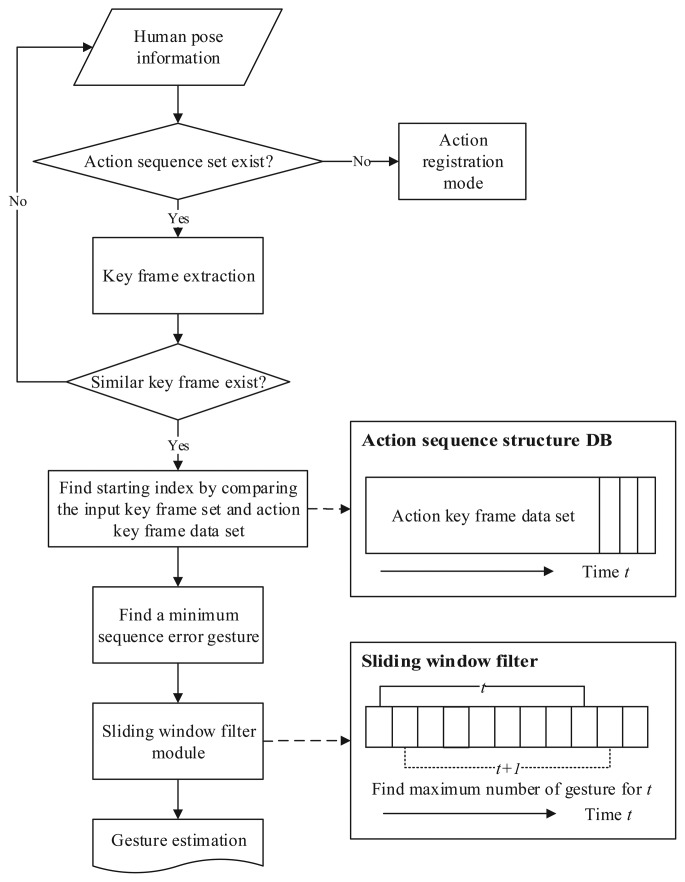
Flow diagram of the proposed gesture recognition method.

The proposed gesture recognition method recognizes gestures by inspecting the pose information from our pose estimation algorithm. The flow diagram of the proposed gesture recognition method is shown in Figure 5. When the pose information is given at each frame, key frames are extracted from the given sequence of pose information. The input pose information is normalized to the specific size for a robust recognition regardless of the body type. The key frames are important frames of the gestures, and they are extracted when the pose information difference between the previous key frame and the current frame is above the pre-defined threshold. Several key frames are extracted until the currently-extracted key frame is similar to one of the previously-generated key frames. The extracted key frames are then compared with key frames of registered gestures. The gesture with the smallest comparison error is chosen as the estimated gesture. The estimated gesture is then passed through the window filter to prevent misrecognition of gestures.

### 3.1. Key Frame Extraction

The human pose estimation result cannot be directly used for gesture recognition, because the joint positions vary depending on the body shape and the distance to the depth sensor. To reduce such effects, the joint information should be normalized to a predefined body size. By applying the normalization process, the joint information fits into a predefined body size.

**Figure 6:**
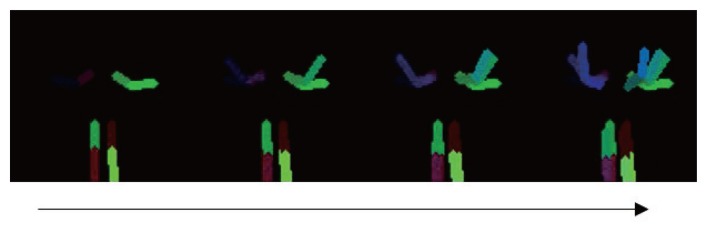
An example of an action sequence.

The key frame extraction is a fundamental part of the proposed gesture recognition method. For each frame, the estimated joint positions are compared with the previous key frame. If the joint position differences between the previous key frame and the current frame are above the pre-defined threshold, the current frame is stored as a key frame. With only consideration of the pose information difference in the key frame extraction process, the same key frames can be extracted for the same gesture regardless of the motion speed. The key frame extraction continues until the currently extracted the key frame is similar to one of the previously-extracted key frames. When the key frame extraction is completed, normalized joint positions of the extracted key frames are stored as an “action sequence.” Figure 6 shows an example of an action sequence. An action sequence contains a sequence of key pose frames for a certain human body motion.

### 3.2. Action Sequence Matching

The gestures are recognized by comparing an input action sequence with the action sequences of registered gestures. A registered gesture contains its name and an action sequence that describes the motions of the gesture. The action sequence in the registered gesture has key frames, and in each key frame, means and standard deviations of joint positions are stored. The registered gesture that gives the smallest comparison error is chosen as the estimated gesture. In the comparison of the input action sequence with registered gesture action sequences, however, the first key frame of the input action sequence does not always match that in the action sequences of the registered gestures, because the key frames are extracted without information about the start and end time of a gesture. We define the start key frame *k_Sg_* in the action sequence of the registered gesture *g* as the keyframe that matches the first key frame of the input action sequence, and it is found by the following equation:

(2) $$k_{S_{g}}=\underset{k\in \left\{0,\ldots ,K-1\right\}}{\arg\min}\frac{1}{N}\sum_{n=0}^{N-1}\frac{{\left(f_{0,n}-m_{g,k,n}\right)}^{2}}{\sigma_{g,k,n}^{2}}$$

where *f*_0,_*_n_* is the *n*-th joint position in the first key frame of the input action sequence, *m_g,k,n_* is the mean of the *n*-th joint position in the *k*-th key frame of the registered gesture action sequence *g*, *σ_g,k,n_* is the standard deviation of the *n*-th joint position in the *k*-th key frame of the registered gesture action sequence *g*, *K* is the total number of key frames in the registered gesture action sequence and *N* is the number of human body joints. Equation (2) delineates that the start key frame in the registered action sequence has the smallest comparison error, which is defined as the mean Mahalanobis distance of joint positions in the first key frame in the input action sequence.

**Figure 7:**
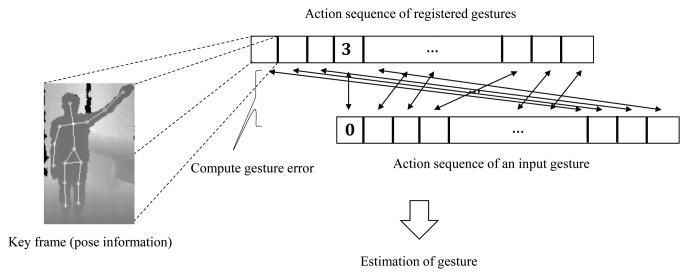
Action sequence matching.

Before the recognition of gestures, gestures that we want to recognize should be defined. In the gesture registration process, a gesture to be registered should be acted several times. In each gesture action, the poses of the gesture should be slightly different so that various movements in a single gesture can be covered. From the generated several action sequences for a single gesture, means and variances of joint positions in each key frame of the gesture are computed. These means and variances of joint positions are used to compute a comparison error with an input action sequence. In addition to the means and variances of the action sequences, maximum comparison errors should be considered to prevent undefined gestures from being recognized as one of the registered gestures and to prevent wrong estimation of gestures. A maximum comparison error of a gesture is set as the smallest comparison error between the other registered gestures. The maximum comparison error of a registered gesture *g* is computed as follows:

(3) $$E_{\max}=\underset{\begin{array}{c} g\prime \in \left\{0,\ldots ,G-1\left(g\prime \neq g\right)\right\},\\ a\in \left\{0,\ldots ,A-1\right\} \end{array}}{\min}\frac{1}{K}\sum_{k=0}^{K-1}\frac{1}{N}\sum_{n=0}^{N-1}\frac{{\left(f_{a,k,n}-m_{g\prime ,k\prime ,n}\right)}^{2}}{\sigma_{g\prime ,k\prime ,n}^{1}},k\prime =\left(k_{S_{g\prime }}+k\right)\mathrm{mod}K$$

where *f_a,k,n_* is the *n*-th joint position in the *k*-th key frame of the *a*-th action sequence from the registered gesture *g*; *m_g′,k′,n_* is the mean of the *n*-th joint position in the *k′*-th key frame of the other registered gestures *g′*; *σ_g′,k′,n_* is the standard deviation of the *n*-th joint position in the *k′*-th key frame of the other registered gesture *g′*; *G* is the number of registered gestures; and *A* is the number of action sequences in the registered gesture *g*. The meaning of Equation (3) delineates that the maximum comparison error of a gesture is the smallest comparison error from every case of comparison between the several action sequences of gesture *g* and the other gestures *g′*. After determining the start key frame in the registered gesture action sequence *g*, the input action sequence and the registered gesture action sequence *g* are compared in a circular manner, as shown in Figure 7. For each registered gesture action sequence, the start key frame is found, and the comparison error with the input action sequence is computed using the following equation:

(4) $$E_{\text{compare}}=\frac{1}{K_{I}}\sum_{k=0}^{K_{I}-1}\frac{1}{N}\sum_{n=0}^{N-1}\frac{{\left(f_{k,n}-m_{g,k^{\prime },n}\right)}^{2}}{\sigma_{g,k\prime ,n}^{2}},k\prime =\left(k_{S_{g}}+k\right)\mathrm{mod}K$$

where *K_I_* is the number of key frames in the input action sequence and *f_k,n_* is the *n*-th joint position in the *k*-th key frame of the input action sequence. Equation (4) indicates that the comparison error of a registered gesture with the input action sequence is the mean Mahalanobis distance of joint positions in the key frames from the input action sequence.

When computation of the comparison error for every registered gesture is done, the gesture with the smallest comparison error is selected as a candidate for a recognized gesture. If the comparison error is below the maximum comparison error of the gesture, the selected gesture undergoes the sliding window filter to minimize misrecognition of gestures. Every time when the input action sequence is estimated, the sliding window filter with a pre-defined length is applied to the sequence. If the count of the same gesture estimation exceeds the pre-defined ratio of the window size, the gesture is accepted as the final gesture recognition result.

## 4. Experiments

In this section, the proposed human pose estimation and gesture recognition method are evaluated. The evaluation is performed on datasets that we created. The quantitative results of the human pose estimation method are given, and the proposed method is compared with OpenNI [22] and Shotton's algorithm [4]. The gesture recognition method is evaluated by defining five gestures and testing the proposed method with 50 datasets for each gesture. The experiments are performed on a PC with an Intel i5 3.0 GHz quad-core CPU with 4-GB RAM. The average computation times of the proposed methods are shown in Table 1. A depth sensor ASUS Xtion pro [21] is used to create the test datasets.

The test datasets are captured with data from a depth sensor Xtion pro installed at about a 2-m height, and the size of the captured images is 320 × 240. The datasets consist of 30 video files containing about 11kframes in total. The ground truth data that contain the joint positions at each frame are created for every test dataset using a commercial motion capture system, iPi Mocap Studio [23]. The iPi Mocap Studio is a scalable markerless motion capture system that uses 3D depth sensors to track human joints and produce 3D animations. This system provides human pose data with centimeter-level accuracy offline.

**Figure 8:**
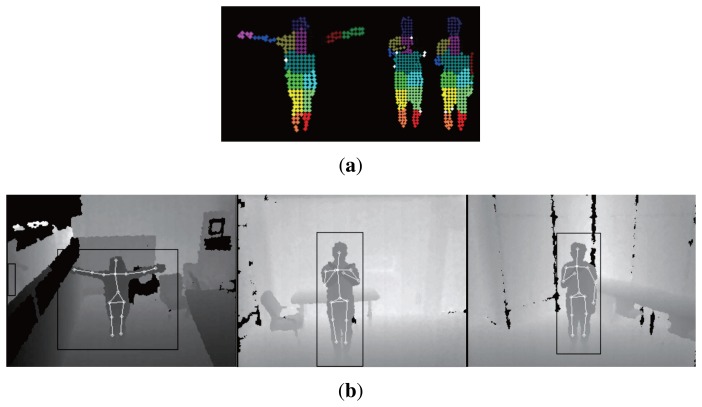
Experimental results of pose estimation. (**a**) Superpixels classification results; (**b**) pose estimation results.

The performance of the proposed human pose estimation method is evaluated by computing the error metrics with ground truth data. The error metrics of the pose estimation algorithm in OpenNI NiTE [22] are also computed and compared with the proposed method. Table 2 shows the error metrics of the proposed method and OpenNI NiTE. The results of OpenNI NiTE show better performance in the accuracy of the pose estimation, but the performance difference of the two methods is small enough. On the other hand, the pose estimation of OpenNI NiTE has the disadvantage that it requires a calibration process that should be performed in the initial stage. Table 3 shows the average initial human pose estimation time of the proposed method and OpenNI NiTE. The initial human pose estimation time is determined by taking the time difference between the first pose estimation and the first input frame with the test dataset. In OpenNI NiTE, the calibration process requires users to take a pre-defined calibration pose for a moment, which sometimes takes more than five seconds. Only after the calibration process, OpenNI NiTE can estimate the poses of users. However, the proposed method does not require a calibration process, and human poses are immediately determined from depth images. These results show that the proposed human pose estimation algorithm is more suitable for a gesture recognition algorithm that requires fast recognition performance. We further tried to compare the human pose estimation efficiency with Shotton's algorithm [4], as shown in Table 4. The comparison was performed by using the performance result of Shotton's paper [4]. The table shows the computational cost for human pose estimation per each frame. According to the comparison result, the computation time of the proposed algorithm requires only about 56% that of Shotton's algorithm. Therefore, the proposed method is more suitable for an implementation in the embedded surveillance system. Figure 8 shows examples of experimental results obtained with the proposed pose estimation method.

**Figure 9:**
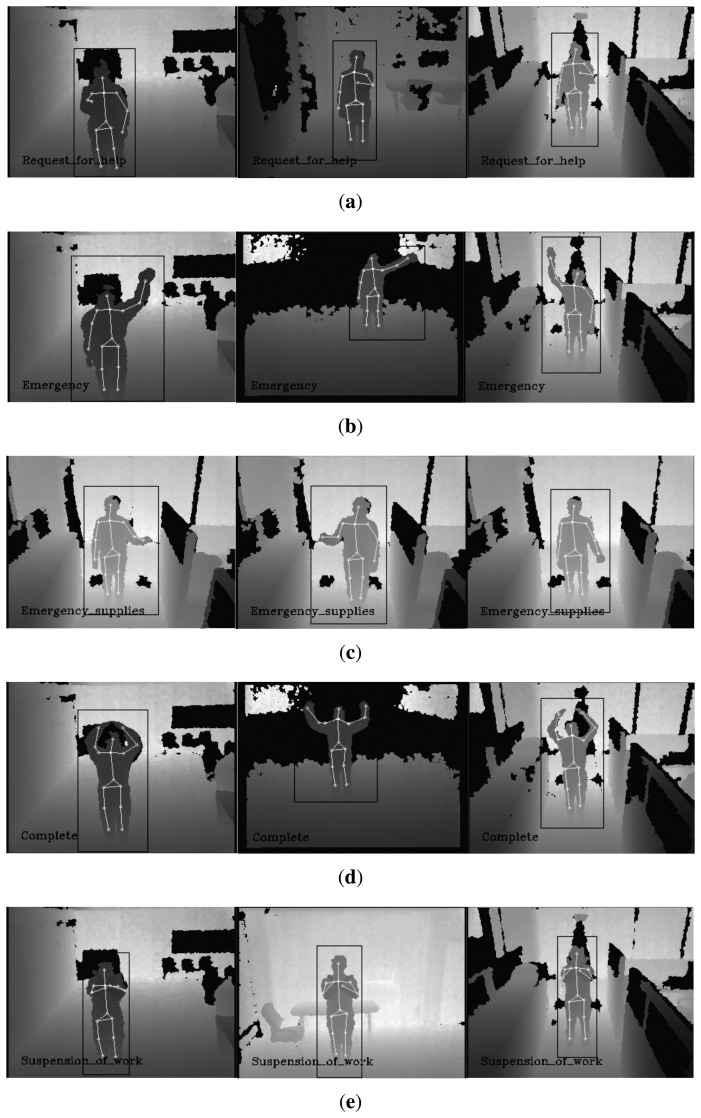
Example experimental results of gesture recognition. (**a**) The experimental results: “request for help”; (**b**) the experimental results: “emergency”; (**c**) the experimental results: “request for emergency supplies”; (**d**) the experimental results: “complete”; (**e**) the experimental results: “suspension of work”.

In the case of the proposed gesture recognition algorithm, we defined five gestures to evaluate the proposed gesture recognition method: “request for help”, “emergency”, “request for emergency supplies”, “complete” and “suspension of work”. “Request for help” is defined as a motion where a person is beating his/her chest with a single hand. “Emergency” is an action of waving a single hand in the air. “Request for emergency supplies” is a motion that resembles someone lifting something with a single hand. The “complete” gesture is making a circle with raised arms. Lastly, “suspension of work” is a motion of crossing arms. We assumed that all gestures were made toward the sensor within the human pose estimation range (±30 degrees). This assumption may not guarantee a good gesture recognition rate depending on the viewing angle of the sensor. This problem, however, can be mitigated by installing multiple sensors for a wider viewing angle. For each gesture, 50 test datasets that recorded the behavior of people with various body types (thin, normal and overweight ratio 1:3:1), genders (male and female ratio 4:1) and clothes (casual, protective clothing and suit clothing) were used to evaluate the performance of the proposed gesture recognition method. If a gesture is recognized correctly once or more than once in a test video, it is counted as a correct recognition; a true positive case. If no gesture is recognized or gestures are recognized incorrectly, it is counted as a false recognition; a false positive case. Table 5 shows the experimental results of the proposed algorithm, and Table 6 shows the false recognition cases of the gestures. The experimental results show that the false recognition rate of ‘emergency supplies’ is higher than that of any other gesture. This result may be attributed to the similarity of the motion of the ‘emergency supplies’ gesture to the partial motion of the other gestures. The experimental results of the proposed gesture recognition method can be improved if we define gestures where motions of the gesture have minimal overlap with each other. Figure 9 shows example experimental results of the proposed gesture recognition method. As a result, using the test datasets that recorded various people, the human pose-based gesture recognition algorithm can recognize the gesture robustly.

## 5. Conclusions

In this work, we proposed a human pose estimation and a gesture recognition method with a depth sensor. In the human pose estimation, joint positions of a human body are estimated only with depth information, and the proposed method can be operated on low-cost platforms without exploiting GPUs. Our pose estimation method is based on per-pixel classification and superpixels. Instead of classifying all of the pixels on a human body, superpixels are generated on a human body, and then, the generated superpixels are classified into one of the body parts. This process greatly reduces the computation time. In gesture recognition, the pose information from the pose estimation method is used to extract key frames. A set of extracted key frames is compared with registered gestures, and a predicted result is passed to the sliding window filter. The key frame extraction enables robust and fast gesture recognition regardless of motion speed. The experimental results show that the proposed human pose estimation and gesture recognition method provide acceptable performance in real environment applications.

The current methods use SVMs for the classification of superpixels, and training data are generated manually. The performance of the current method can be improved by dealing with the following topics.

- Employing other classification algorithms for body part classification, such as a deep learning method.
- Applying kinematic constraints of a human body in pose estimation.
- Estimating human orientation to compensate for human orientation change in the human pose estimation process.
- Using better feature information for body part classification.

## Figures and Tables

**Table 1:** Average computation times of the proposed methods.

	**Pose estimation**	**Gesture recognition**	**Total frame rate**
Time	65 ms	less than 1 ms	15 fps

**Table 2:** Experimental results of pose estimation. L, left; R, right.

**Body part**	**Mean (mm)**		**SD (mm)**	

	**Proposed method**	**OpenNINiTE**	**Proposed method**	**OpenNI NiTE**
Head	26.0	26.0	25.2	15.5
Neck	53.3	37.5	28.4	26.4
Torso	75.3	121.0	31.7	32.8
L Shoulder	41.2	45.7	21.9	24.6
L Elbow	86.2	80.1	69.2	66.4
L Hand	199.4	128.2	332.6	156.1
R Shoulder	49.6	34.3	20.9	23.2
R Elbow	87.7	68.9	61.9	52.2
R Hand	190.8	97.4	306.7	120.2
L Hip	44.2	34.9	29.0	27.2
L Knee	133.0	44.3	44.3	24.6
L Foot	114.0	68.7	28.6	41.3
R Hip	58.2	43.9	23.8	29.6
R Knee	130.0	52.6	47.0	26.2
R Foot	96.8	81.6	28.0	74.4

Average	92.3	64.3	73.3	48.8

**Table 3:** Average initial human pose estimation time (unit: ms).

	**Proposed Method**	**OpenNI NiTE**
Time	67.0	2413.1

**Table 4:** Computational cost of human pose estimation (unit: GFlops).

	**Proposed method**	**Shotton's Algorithm [4]**
Computational cost for each frame	0.81	1.44

**Table 5:** Experimental results of gesture recognition.

**Gesture**	**Recognition**	**False Recognition**	**Recognition Rate**	**False Recognition Rate**
“Request for help”	47	11	94.0%	5.5%
“Emergency”	43	4	86.0%	2.0%
“Request for emergency supplies”	49	24	98.0%	12.0%
“Complete”	46	7	92.0%	3.5%
“Suspension of work”	47	1	94.0%	0.5%

Total	232	47	92.8	4.7

**Table 6:** False gesture recognition results.

**Gesture Dataset**	**False Recognition Cases**				
	**“Request for Help”**	**“Emergency”**	**“Request for Emergency Supplies”**	**“Complete”**	**“Suspension of Work”**
“Request for help”	None	0	0	1	0
“Emergency”	0	None	5	0	0
“Request for emergency supplies”	1	4	None	0	0
“Complete”	0	0	5	None	1
“Suspension of work”	10	0	14	6	None
Total	11	4	24	7	1
